# Ambiguity in Tactile Apparent Motion Perception

**DOI:** 10.1371/journal.pone.0152736

**Published:** 2016-05-12

**Authors:** Emanuela Liaci, Michael Bach, Ludger Tebartz van Elst, Sven P. Heinrich, Jürgen Kornmeier

**Affiliations:** 1 Institute for Frontier Areas of Psychology and Mental Health, Freiburg, Germany; 2 Eye Center, Medical Center, University of Freiburg, Freiburg, Germany; 3 Clinic for Psychiatry and Psychotherapy, Medical Center, University of Freiburg, Freiburg, Germany; 4 Faculty of Medicine, University of Freiburg, Freiburg, Germany; Ecole Polytechnique Federale de Lausanne, SWITZERLAND

## Abstract

**Background:**

In von Schiller’s Stroboscopic Alternative Motion (SAM) stimulus two visually presented diagonal dot pairs, located on the corners of an imaginary rectangle, alternate with each other and induce either horizontal, vertical or, rarely, rotational motion percepts. SAM motion perception can be described by a psychometric function of the dot aspect ratio (“AR”, i.e. the relation between vertical and horizontal dot distances). Further, with equal horizontal and vertical dot distances (AR = 1) perception is biased towards vertical motion. In a series of five experiments, we presented tactile SAM versions and studied the role of AR and of different reference frames for the perception of tactile apparent motion.

**Methods:**

We presented tactile SAM stimuli and varied the ARs, while participants reported the perceived motion directions. Pairs of vibration stimulators were attached to the participants’ forearms and stimulator distances were varied within and between forearms. We compared straight and rotated forearm conditions with each other in order to disentangle the roles of exogenous and endogenous reference frames.

**Results:**

Increasing the tactile SAM’s AR biased perception towards vertical motion, but the effect was weak compared to the visual modality. We found no horizontal disambiguation, even for very small tactile ARs. A forearm rotation by 90° kept the vertical bias, even though it was now coupled with small ARs. A 45° rotation condition with crossed forearms, however, evoked a strong horizontal motion bias.

**Discussion:**

Existing approaches to explain the visual SAM bias fail to explain the current tactile results. Particularly puzzling is the strong horizontal bias in the crossed-forearm conditions. In the case of tactile apparent motion, there seem to be no fixed priority rule for perceptual disambiguation. Rather the weighting of available evidence seems to depend on the degree of stimulus ambiguity, the current situation and on the perceptual strategy of the individual observer.

## Introduction

The capacity of our sensory organs is restricted and thus the availability of the information from the world around us. Our perceptual system needs to make sense of this incomplete and to varying degrees ambiguous information in order to construct the most coherent interpretations (e.g. [1]). Ambiguous figures, like the famous Necker cube [2], are extreme cases of sensory ambiguity. The corresponding percepts are only temporarily stable and alternate between different but equally probable interpretations, even though the stimulus, and thus the retinal input, remain constant. Multistability can be evoked by a variety of ambiguous figures, involving depth ambiguity e.g. [2], figure/ground organization (e.g., Rubin’s face/vase figure [3]), motion ambiguity (e.g., von Schiller’s Stroboscopic Alternative Motion stimulus, “SAM” [4]), or even semantic ambiguity (e.g., Boring's Old/Young woman [5]). Another class of multistable perception is binocular rivalry, when different images are presented to the two eyes and perception alternates spontaneously between the two images [6,7]. All cases of multistability share some common features: (*i*) perceptual alternations show a stochastic pattern and can be modeled by gamma distributions [8,9] and (*ii*) perceptual alternations can be influenced volitionally, e.g. [10]. Several authors have thus postulated that sensory ambiguity is processed at high-level/cognitive units (e.g., the seminal review [11]). Multistable perception has been studied predominantly in the visual modality. However, very similar effects have been described in other sensory modalities, like audition (e.g. [12,13]) and olfaction (e.g. [14]). Similarity of ambiguity features across sensory modalities may further support this high-level/cognitive explanations, mentioned above.

Studies of ambiguity in the tactile modality used tactile apparent motion stimulus variants. Harrar and Harris [15] applied a tactile version of the visual “Pikler-Ternus illusion” [16]: The sequential presentation of frames composed of three dots can be perceived as either group or element motion, as a function of the inter stimulus interval between frames (for a demonstration see Ternus). Like the visual Ternus, the tactile variant was ambiguous with spontaneous alternations between group and element motion, however, with lower rates of perceptual alternations (e.g. [17]).

Von Schiller’s ambiguous stroboscopic alternative motion (SAM) stimulus [4], (also known as the “apparent motion quartet” [18]), is another apparent motion stimulus that can be realized both in the visual and tactile modalities and will be used in the present study. The visual SAM consists of two diagonal pairs of dots alternately presented at the corners of an invisible rectangle. The alternating appearance of the dot pairs integrates into a coherent percept of apparent motion, whereby we perceive two dots moving in opposite directions. The perceived motion direction is a function of the rectangle’s *aspect ratio (“AR”)*, i.e. the ratio between horizontal and vertical distances of the four dots. Observers perceive horizontal motion with small horizontal and large vertical dot distance (AR << 1), and vice versa. In rare cases observers also report rightwards or leftwards rotation, e.g. [19]. At a certain AR perception becomes unstable and spontaneously changes between motion directions (for an online demonstration see SAM). In particular, the dependence of the perceptual outcome on the AR can be modeled by a psychometric function, e.g. [20]. Interesting in this context is a vertical perceptual bias of the SAM stimulus: At an AR = 1 (when vertical and horizontal dot distances are equal) we would expect parity of percepts but typically find a bias towards vertical motion [21,22].

In the tactile version of the SAM stimulus the observers are stimulated with pulses of four vibrotactile stimulators. Carter et al. [18] have demonstrated perceptual instability during tactile SAM stimulation on the finger tips. In contrast to the visual modality, however, changing the AR of the tactile stimulators had no significant perceptual disambiguation effect in their study. A major difference between the visual and tactile stimulation is that the spatial location of a tactile stimulus can be mapped to a somatotopic/skin-based *endogenous reference frame*, in addition to a space-based/world-based *exogenous reference frame*, shared by both vision and touch. In the endogenous reference frame, the stimuli are encoded in relation to one’s own body surface, whereas the exogenous reference frame refers to the external (world) representation of stimuli. Conflicting information from the two reference frames might explain the missing disambiguation for extreme AR values in the tactile SAM.

An interesting variant of the tactile SAM occurs when the two pairs of vibrotactile stimulators are placed at corresponding fingers of the left and right hand [23,24]. A change of the within-finger distance of the vibrotactile SAM stimulators then entails a change in both endogenous and exogenous reference frames, whereas a change of the between-limb distance changes the position of the stimulators only in the exogenous reference frame. This might allow disentangling the influences of the exogenous and the endogenous reference frames on tactile motion perception. Conrad et al. [23] attached two coin-sized vibrators, with small LEDs on top, to each index finger of their participants. Across four different conditions they either used unimodal visual or tactile stimulation and either spatially congruent or incongruent bimodal stimulation with three different finger distances. Overall, they found more stable motion perception in the tactile modality and with congruent bimodal stimulation compared to pure visual stimulation. Furthermore, the perceived motion direction was a function of finger distance with “horizontal”/ between-finger motion for small finger distances (AR = 0.5), “vertical”/ within-finger motion for larger finger distances (AR = 2) and most unstable percepts with AR = 1 (i.e. equal horizontal and vertical stimulator distance). The disambiguated tactile SAM stimuli (small and large finger distances), however, produced less clear perceptual biases (≈ 60%) than the visual SAM stimulus (close to 100% as reported in [20]). A similar pattern of results was found in Vitello [24], who also used tactile stimulators on the index fingers and varied finger distances between five different AR values (0.25, 0.5, 1, 2 and 4) in the tactile SAM stimulus. He found an increasing bias towards between-finger (horizontal) motion perception with smaller between-finger distances, but no clear bias towards within-finger (vertical) motion for larger between-finger distances. Further, the amount of tactile bias in Vitello’s data again did not exceed 60% even with more extreme ARs, cf. [23]. Vitello’s tactile data displayed a linear relation between finger distance and perceptual outcome, which is in contrast to the sigmoidal shape observed in the visual modality [20].

In summary, apparent motion stimuli, e.g. von Schiller’s SAM, are well suited to compare perceptual processing of visual with tactile ambiguity, because highly similar stimuli can be used in the two modalities. Studies from the visual modality show clear stimulus disambiguation and perceptual stabilization of horizontal and vertical motion for small and large SAM ARs respectively and perceptual instabilities at intermediate ARs, together with a strong bias towards vertical motion at an AR = 1. The few previous studies with a tactile variant of the SAM stimulus provide a less clear picture. Changing the AR of the tactile SAM had only a weak stimulus disambiguation effect, compared to the visual modality, without the expected sigmoidal shape and no clear vertical bias at AR = 1. Ambiguity seems to be processed differently in visual and tactile modalities, which argues against the postulated role of central high-level factors beyond sensory modalities during ambiguity resolution, e.g. [11].

In the present study we focus on two aspects:

(1) One potential explanation for the difference in influence of AR on motion disambiguation may be a different spatial scaling in the tactile compared to the visual modalities. In order to test this, we applied tactile SAM stimuli with a larger range of ARs than previous studies. Tactile perceptual disambiguation close to 100% at more extreme values of ARs (larger and smaller) would point to overall cross modal similarities in motion disambiguation but different spatial scales for motion gestalt integration.

(2) Another potential explanation for the difference in influence of AR on motion disambiguation between the two modalities may be an interplay of exogenous with the endogenous reference frames during the processing of tactile information while only one (exogenous) reference frame is available in the visual modality. In a series of experiments we thus varied the mapping between exogenous and endogenous reference frames by forearm rotation and studied how this affects the disambiguation of ambiguous tactile motion information.

Sensory information is to varying degrees ambiguous. Its disambiguation is a major challenge of our perceptual system, even though we do not notice this in our every day experience. The present study aims to further our understanding of modality-specific and modality-independent aspects of sensory disambiguation, and in particular how information from different reference frames are integrated.

## Methods

### Participants

All participants were naive as to the specific experimental question and gave their written informed consent. None of them reported any history of neurological disease. Visual acuity was tested with the Freiburg Visual Acuity Test [25,26] and all participants had a normal or corrected-to-normal vision. The study, consisting of five experiments, was approved by the ethics committee of the University of Freiburg and performed in accordance with the ethical standards laid down in the Declaration of Helsinki [27].

### Experiments

The present study consisted of five experiments. In Experiments 1 and 2 we applied a tactile variant of the visual SAM to the participants’ forearms, placed straight parallel on the table in front. We studied tactile SAM motion perception as a function of a large range of aspect ratios. In Experiment 3 we compared two configurations of tactile SAM perception, one with straight parallel and one with forearms rotated by 90°, in order to disentangle the contribution from endogenous and exogenous reference frames. In Experiments 4 and 5 we rotated the participants forearms by 45°, resulting in crossed forearms, and compared the perceptual results from two different tactile SAM stimulation protocols. Experimental details are provided in the respective sections.

#### Vertical visual vs. “vertical” tactile motion

**Fig 1:**
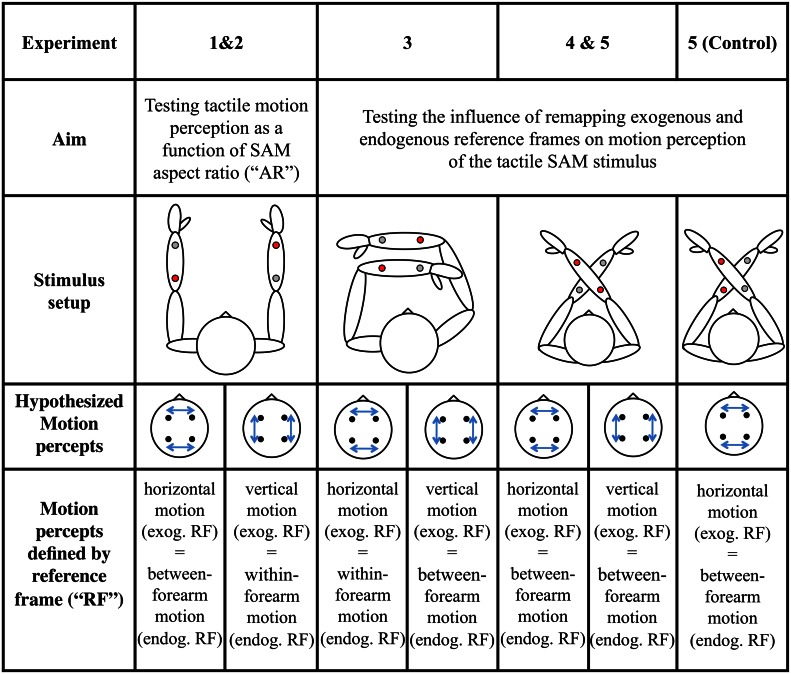
Overview of the tactile Experiments. Schematic illustration of the stimulus setups, hypothetical motion percepts and their description with respect to the two different reference frames (RF).

In the visual modality perceived “vertical motion” was defined as motion between the upper and lower right (or left) corners of the imaginary rectangle, e.g. [19]. In the tactile modality we adopted the definitions from previous studies on the tactile SAM variants of perceived “vertical motion” as motion in depth to and away from the observer’s body along the arms that were positioned on a table in front of the participants [23,24] (see also Fig 1). This can be motivated by the following considerations: At the earliest step of vision, a three dimensional world is projected onto two-dimensional retinae. As a consequence, up and down motion as well as motion to and away from the body are projected onto the same axis. The depth axis in the tactile experiment and the vertical axis in typical visual SAM experiments may thus be represented in the same way. Despite this, the results of the present experiments have to be regarded under the caveat that vertical visual motion is located in another 3D plane than vertical tactile motion.

### Data analysis

We calculated generalized linear regression analyses with a binomial family function and a logit link function in order to test relations between the direction of motion perception (dependent variable) and horizontal and vertical stimulator distances (independent variables).

We applied non-parametric statistical permutation tests [28] analogous to classical t-tests in order to test deviations of motion perception from chance level (0.5). The basic idea of permutation tests is to generate reference distributions out of the measured data by randomizing their relation to experimental conditions. Correction for multiple testing was applied with Holm’s variant of the Bonferroni correction [29]. The Holm-procedure starts by ordering the p-values from all executed tests. It then compares the smallest p-value with an alpha level corrected by the number n of individual tests, as suggested by Bonferroni. It continues by comparing the subsequent p-values with step-wise adjusted alphas (e.g. second smallest p-value compared to an alpha level corrected by n-1, etc.).

## Experiment 1: Tactile Analogue of a Typical Visual SAM Experiment

### Background

We applied a tactile version of the SAM stimulus with varying ARs and studied tactile apparent motion percepts as a function of AR.

### Participants

Nine participants (6 males, 3 females; age range = 20–27, mean age = 23 years) participated in Experiment 1. All participants were right-handed.

### Tactile SAM stimuli

For tactile SAM stimulation four SHIVR HEK-100 stimulator kits (Midé Technology Corp., Medford, MA, USA) were used. Each consisted of a piezoelectric tactor and the respective driver electronics, into which sequences of 2-V pulses were fed to produce vibrations. These voltage pulses were controlled through custom software based on Igor Pro (Wavemetrics, Inc., Lake Oswego, OR, USA) running on a Mac Mini computer. A single vibration burst consisted of 30 pulses at a frequency of 100 Hz, each lasting for 5 ms and being followed by a gap of 5 ms. Each stimulator had a diameter of 0.32 cm.

### Paradigm

The participants were seated with their arms on a table placed in front of them. The arms were oriented in parallel to each other in straight-ahead direction. Two stimulators were fixed at each of the observer’s forearm. The four stimulators were positioned in parallel within and between the arms at the four corners of an imaginary rectangle. We defined distances of stimulator locations in relation to the total forearm length of the individual participant in order to control for inter-individual variability in forearm length. The two stimulators of each stimulator-pair were placed at the center of the two forearms at a distance of 50% of the participants total forearm length and seven different between-forearm distances ranging from 20% to 200% of the participants total forearm length in steps of 33.33%. This resulted in 7 different tactile SAM stimuli, corresponding to the following ARs: 0, 0.67, 1.34, 2, 2.67, 3.34 and 4. All ARs of the tactile SAM stimuli were calculated with respect to the exogenous reference coordinates. An AR of 0 corresponds to a configuration where the two forearms touch each other. However, the stimulators from one forearm never touched the neighboring forearm and all participants had clear antiparallel motion percepts in this configuration.

All participants were blindfolded during the tactile SAM experiment because normally vision dominates the other senses in spatial processing [30]. In particular, Vitello [24] found visual influence on the perception of the ambiguous tactile SAM variant. In addition, our participants wore headphones during the experiment, listening to music in order to mask the vibration sound of the stimulators.

The tactile SAM stimuli were presented in a sequence of four frames. Each frame contained one pair of two diagonal vibrators pulsing for 300 ms and was followed by an inter-pulse interval of 200 ms per frame. Each vibrator pair was thus active twice within a sequence and was followed by a response interval of 3000 ms. During the latter, participants indicated horizontal or vertical motion percepts by pressing one of two different pedals of a three-pedal foot device (Scythe USB_3FS-2 triple foot switch). In the case of other percepts, the participants pressed a third pedal. Any pedal press terminated the response interval and started the next SAM sequence. Preliminary tests suggested that operating 5 keys of the foot device could potentially overburden the participants. Given the rare occurrence of rotational motion, we therefore decided to use only 3 switches. None of the participants reported any case of rotational motion perception in the tactile experiments. Whenever participants responded outside the response interval (i.e. too early or too late), this SAM sequence was repeated. The starting stimulator pair was randomized between sequences. Before executing the experiment, all participants were trained to associate each key with the corresponding tactile motion direction.

Some participants reported perceiving dot-like objects jumping back and forth either along the forearms (within-forearm or vertical motion) or between the forearms (between-forearm or horizontal motion, Fig 1). Other participants reported perceptions of an imaginary rod, whose partial back and forth rotation induced tactile stimulations on the forearms. In this case they perceived the front end (and likewise the back end) of the rod oscillating either on one forearm (vertical motion) or between the forearms (horizontal motion).

**Fig 2:**
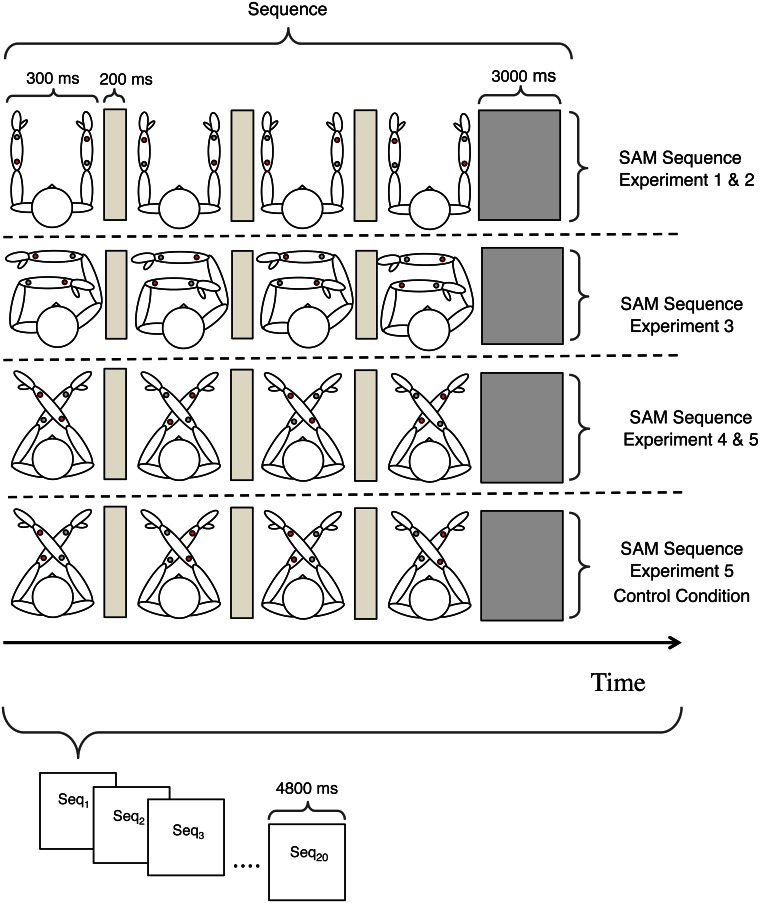
Tactile SAM Paradigm. Within each experimental block, 20 SAM sequences (Seq_i_) were presented for 4800 ms each and participants indicated their motion percepts at the end of each sequence. AR was kept constant within SAM sequences and blocks but changed randomly between blocks. The number of ARs, and thus the number of blocks, varied between experiments. Experimental blocks with specific SAM ARs occurred in random order in the first half of the experiment. This random order was reversed in the second half of the experiment resulting in 40 sequences per AR in total. Small red and white circles: Respectively active and inactive tactile stimulators.

One specific SAM sequence was repeated 20 times within one experimental block. AR and thus SAM stimulator attachment was changed between experimental blocks. The order of the 7 experimental blocks, each with a specific SAM stimulus, was randomized in the first half of the experiment. The blocks were repeated in reverse order in the second half of the experiment (ABBA scheme). This resulted in 40 presentations of each of the seven SAM stimuli (see Fig 2).

### Results

The mean percentage of percepts other than vertical or horizontal motion across conditions was 4 ± 2%.

The generalized linear model indicated no effect of horizontal and vertical stimulator distance on the direction of motion perception.

**Fig 3:**
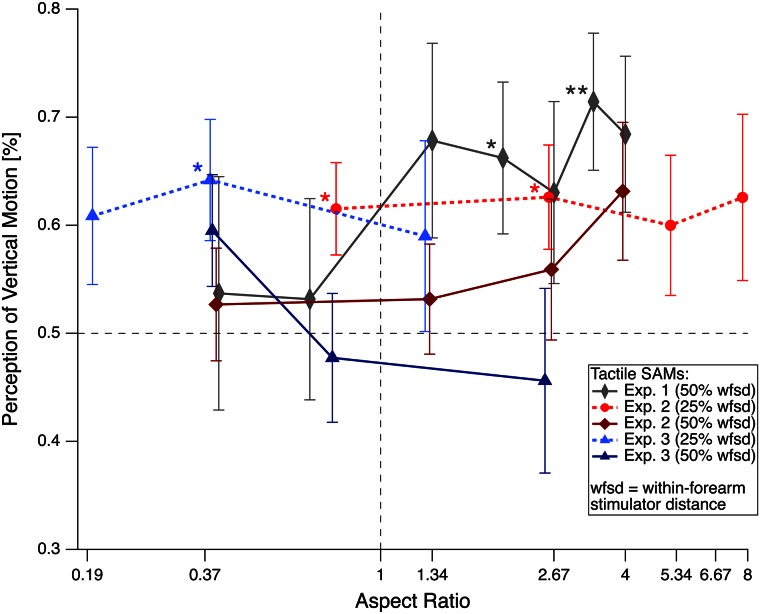
Results from Experiments 1–3. Percentage of vertical motion percepts as functions of the stimulators’ AR. Symbols are grand means ± SEM. Within-forearm dot distances were 50% total forearm length (grey, dark blue and red traces) and 25% (light blue and red traces). The grey trace depicts results from Experiment 1, the red traces depict results from Experiment 2 and the blue traces results from Experiment 3. The horizontal dotted line indicates chance level, i.e. equal probability for vertical and horizontal motion percepts. In contrast to the visual SAM we here see very little influence of AR on perceptual dominance—in the visual modality this figure would resemble a psychometric function, running from bottom left to top right. Stars indicate statistical significance (* p < 0.05; ** p < 0.01). [wfsd = within-forearm stimulator distance].

Individual randomization tests for difference of perceived motion direction from chance level indicated a bias towards vertical (i.e. within-forearm) motion perception with aspect ratios larger than one. After correction for multiple testing this bias remained significant for ARs of 2.0 and 3.34 (see Table 1). However, this vertical bias was much weaker (roughly 70%, see Fig 3, grey trace) than the vertical bias for large ARs as reported from the visual modality (close to 100%, e.g. [31]). We did not observe a bias towards horizontal motion perception, not even for the smallest horizontal stimulator distances (54% vertical motion percepts, p = 0.35 for the test against chance level). The statistical results from Experiment 1 are listed in Table 1.

**Table 1:** Statistical Results from Experiment 1.

Aspect Ratio (Horiz./between- forearm stimulator distance in % forearm-length)	p-Value
0.4 (20%)	0.35
0.67 (33%)	0.36
1.34 (67%)	0.035
2.0 (100%)	0.0055*
2.67 (133%)	0.075
3.34 (167%)	0.0004**
4.0 (200%)	0.014

Significance level after correction for multiple testing:

* p < 0.05;

** p < 0.01

### Discussion

Perception of the tactile SAM stimuli showed the following similarities to the visual modality. First both the visual and the tactile SAM stimuli induced apparent motion perception. Second, this apparent motion perception was ambiguous in a certain range of stimulus parameters. Third, taking into account that during visual perception a three-dimensional world is projected onto two-dimensional retinae and labeling both the visual up and down motion and the tactile motion in depth away and towards the body as “vertical motion”, both modalities showed a perceptual bias towards vertical motion.

Despite these similarities there were also obvious differences between the visual and the tactile modalities. First, apparent motion perception in the tactile modality was overall less stable than in the visual modality—it stayed ambiguous even for large ARs (up to 1.3) and only became biased with ARs of 2 and larger (see Fig 3, grey trace). Second, we did not find any bias towards horizontal (between forearms) tactile motion perception, even for the smallest between-forearm stimulator distance when grouping by proximity would predict horizontal motion perception [32]. Similar deviations from the proximity rule in the tactile modality have been reported in other studies [18,33]. This is in stark contrast with the visual modality, where even small ARs elicited close to 100% horizontal motion perception (e.g. [20, 31]).

#### Possible explanations of extended perceptual ambiguity and the vertical motion bias in the tactile modality

As noted before, one obvious difference between modalities is the involvement of *two* different representational systems in the tactile modality but only *one* in the visual modality. Specifically, vertical tactile motion direction can be organized by referring to an endogenous, somatotopic reference frame. In this way an imaginary object jumps back and forth between two neighboring points of the body—in the present case the forearm. These two points may then be represented by two adjacent neural assemblies in the somatotopically organized somatosensory cortex. Proximity of the two cortical representations may thus enter as input from the endogenous reference frame to the perceptual motion interpretation. In endogenous reference frame coordinates the vertical (within-forearm) distance between stimulators is always smaller than the horizontal distance. This is because the representations of the stimulators from different forearms are in the somatosensory cortices of the contralateral hemispheres. SAM ARs (= horizontal distance / vertical distance) expressed in endogenous reference frames are thus always larger than 1, supporting the vertical motion interpretation.

Concurrently, tactile motion perception can also be constructed by referring to an exogenous space-based or world-based reference frame. The position of the forearm, the position of the stimulators on the forearm, and the back and forth jumps of the imaginary object can thus also be described in exogenous world-based coordinates. Here, the horizontal stimulator distance depends on the arm positions and small arm distances lead to ARs between 0 and 1, thus supporting a horizontal motion interpretation. For large arm distances, in contrast, ARs are greater than 1, supporting a vertical motion interpretation.

The information from the two reference frames needs to be conciliated in order to construct a stable and reliable motion percept. For large forearm distances the analysis outputs from both reference frames support a vertical motion interpretation. This may explain the observed tendency for vertical motion interpretations with large ARs. For small forearm distances, in contrast, information from the two reference frames support opposite interpretations evoking perceptual instability for a broad range of smaller ARs and, in particular, preventing a bias towards horizontal motion perception.

An obvious question remains, however, with this explanation: Why is the vertical bias for large ARs so weak in the tactile compared to the visual modality?

## Experiment 2: Increasing the Tactile AR Range

### Background

Tactile SAM stimuli stayed ambiguous for a large range of ARs, which is in strong contrast to what is known from the visual modality. Potential differences in tactile and visual scaling may require more extreme AR values for tactile SAM disambiguation. In Experiment 2 we thus explored whether a further increase of AR would increase the vertical motion bias towards more stable motion percepts, comparable to the visual modality. Since we are anatomically restricted with respect to a further increase of the horizontal stimulator/forearm distances, we increased ARs by simply decreasing the vertical stimulator distance.

### Participants

Fifteen participants (8 males, 7 females; age range = 20–27, mean age = 23.7 years) participated in Experiment 2. All but one were right-handed. Five participants had participated in Experiment 1.

### Paradigm

Experiment 2 was identical to Experiment 1 with two changes:

(1) We only used four between-forearm distances: 20%, 66%, 133% and 200% of the participant’s total forearm length. (2) We combined these four between-forearm distances with two within-forearm distances: 25% and 50% of the participant’s total forearm length. This resulted in 8 combinations of within- and between-forearm distances, corresponding to the following 8 ARs: for 25% within-forearm distance, 0.8, 2.67, 5.32, 8; for the 50% within-forearm distance, 0.4, 1.34, 2.67, 4. Each AR was presented two times (i.e. in two blocks; see Fig 2). For technical reasons (recording failure) we had to dismiss one repetition for one participant for one combination.

### Results

The mean percentage of percepts other than vertical or horizontal motion across conditions was 5 ± 2%.

For the 50% vertical (within-forearm) stimulator distances only large horizontal (between-forearm) stimulator distances showed a weak perceptual bias towards vertical motion perception (see Table 2 and the dark red trace in Fig 3). For the 25% vertical (within-forearm) stimulator distance the vertical motion bias is more obvious and seems to be present for all horizontal stimulator distances, although only the two smallest remained significant after correction for multiple testing (Table 2 and the light red trace in Fig 3). Again, this vertical bias in the tactile modality is weak (maximal 62% vertical motion perception).

**Table 2:** Statistical Results from Experiment 2.

between-forearm stim. dist. [%]	within-forearm stim. dist. [%]	Aspect Ratio	p-Value
20	50	0.4	0.31
67	50	1.34	0.27
133	50	2.67	0.19
200	50	4.0	0.022
10	25	0.8	0.0054*
67	25	2.67	0.0072*
133	25	5.32	0.067
200	25	8.0	0.056

Significance level after correction for multiple testing:

* p < 0.05;

Stimulator distances expressed as % total forearm length

### Discussion

In Experiment 2, we tested whether a further increase of AR might further stabilize the weak vertical motion bias from Experiment 1 to values comparable to the visual modality.

For the 50% within-forearm/vertical stimulator distance AR values between 0 and 4 stayed in the range of Experiment 2 and motion perception remained largely ambiguous, close to a value of 0.5 (equal probability for horizontal and vertical motion perception). Only at AR = 4 we found a weak tendency for vertical motion bias, which however did not remain significant after multiple testing correction according to Holm (see Table 2 and dark red trace in Fig 3).

The 25% within-forearm stimulator distance extended the range of ARs to 8. With smaller within-forearm stimulator distances the vertical motion bias seemed to occur already for smaller ARs. However it stayed at a weak level of around 65%, which seemed to be the maximal available degree of perceptual disambiguation of apparent motion information for the tactile modality—at least in the so far tested range of ARs (see Table 2 and light red trace in Fig 3).

In summary, increasing AR by decreasing vertical (within-forearm) stimulator distance seemed to promote the occurrence of a vertical motion bias but doubling AR did not further increase its intensity.

## Experiment 3: Remapping Endogenous and Exogenous Reference Frames

### Background

Perception of ambiguous tactile SAM stimuli remained highly ambiguous, even for very small ARs. SAM motion perception was only weakly disambiguated towards vertical motion and only for very large ARs up to 8. In Experiment 3 we tested whether the weak vertical motion bias for large ARs and the missing horizontal bias for small ARs result from confirmative and contradictory evidences from the two reference frames. In Experiment 3, participants’ forearms were rotated by ±90° in order to change the mapping of endogenous to exogenous reference axes. After this forearm rotation, vertical motion (exogenous reference frame) was related to between-forearm motion (endogenous reference frame) and vice versa (Fig 1). If our results can be explained by the interaction of reference frame information, this realignment of reference frames should markedly change the pattern of results. At small ARs, we should expect to find higher likelihood for horizontal motion because small horizontal stimulator distances in exogenous coordinates correspond to small distances in endogenous coordinates, and large vertical stimulator distances in exogenous coordinates correspond to large distances in endogenous coordinates. At large ARs, in contrast, we would expect contradictory evidence, because small vertical stimulator distances in exogenous coordinates correspond to large distances in endogenous coordinates. According to these considerations we would expect a (weak) horizontal motion bias, but no more vertical bias.

### Participants

Twelve participants (7 males, 5 females; age range 20–28 years, mean age = 24.4) participated in Experiment 3. All but one of the participants were right-handed. One participant of Experiment 3 had participated also in Experiment 1, whereas three had participated in Experiment 2.

### Paradigm

Experiment 3 was identical to Experiment 2 with the following changes:

(*i*) The forearm positions were rotated by ±90° compared to Experiments 1 and 2. This resulted in forearm orientations orthogonal to the straight-ahead direction (see Fig 1). (*ii*) We kept the two within-forearm stimulator distances from Experiment 2 but used only the first three between-forearm distances (20%, 66% and 133%), leaving out the fourth one (200%), which is anatomically impossible in this configuration. This resulted in 6 combinations of within- and between-forearm distances (corresponding to the following 6 ARs: for 25% within-forearm distance, 0.19, 0.37, 1.25; for the 50% within-forearm distance, 0.38, 0.75, 2.5). Each AR was presented twice, i.e. in two blocks.

### Results

The mean percentage of percepts other than vertical or horizontal motion across conditions was 3.0 ± 1.4%.

For the 50% vertical (within-forearm) stimulator distances only large horizontal (between-forearm) stimulator distances—in this case corresponding to small ARs—showed a weak tendency for a perceptual bias towards vertical motion perception (see Table 3 and dark blue trace in Fig 3), which was insignificant after multiple testing corrections. For the 25% vertical (within-forearm) stimulator distance, the vertical bias in motion perception was again observable only for large horizontal (between-forearm) stimulator distances (i.e. small ARs). It remained significant for an AR of 0.37 after correction for multiple testing (Table 3 and light blue trace in Fig 3).

**Table 3:** Statistical Results (p values) from Experiment 3.

between-forearm stim. dist. [%]	within-forearm stim. dist. [%]	Aspect Ratio	p-value
20	50	2.5	0.7
67	50	0.75	0.63
133	50	0.38	0.042
20	25	1.25	0.16
67	25	0.37	0.0076*
133	25	0.19	0.051

Significance level after correction for multiple testing:

*p < 0.05;

Stimulator distances expressed as % total forearm length

### Discussion

In this experiment we rotated the forearms by ±90°, with an additional realignment of exogenous and endogenous reference frames and in particular a reassignment of within- and between-forearm motion to horizontal and vertical motion. We had expected opposite results compared to Experiments 1 and 2 with a horizontal motion bias for small ARs and ambiguous percepts for large ARs but no vertical bias. However, we found again a weak perceptual bias towards vertical motion perception, which corresponds in this experiment to between-forearm motion. Nonetheless, the effect is smaller in Experiment 3 compared to Experiments 1 and 2. Perceptual data from the visual modality predict a clear horizontal motion bias at such a small AR value.

These results are in contrast to our above hypothesis, which makes explanations based on the interaction of reference frames less plausible. One possible alternative explanation of these unexpected results may be the existence of general preference for vertical motion (described in exogenous coordinates). In this case, the information from the endogenous reference frame may have been underweighted during the perceptual disambiguation process in Experiment 3 and thus forearm rotation may have been largely ignored.

## Experiment 4: Crossing Forearms, Variant 1

### Background

The tactile motion perception was ambiguous for a broad range of SAM ARs with only a weak vertical but no horizontal bias. Remapping of endogenous and exogenous reference frames by 90° forearm rotation did not change the pattern of results. To further test the role of the reference frame interaction on tactile motion perception we tested a condition with 45° forearm rotation.

### Participants

11 participants (6 males, 5 females; age range 23–27, mean age = 26.5) participated in Experiment 4. All but one of the participants was right-handed. One participant had participated in Experiment 2 and seven had participated in Experiment 3.

### Tactile SAM stimuli and Paradigm

Experiment 4 was similar to Experiment 1 with the following exceptions:

(*i*) The position of the arms was rotated by 45° and thus crossed compared to the forearm posture in Experiment 1, with the left forearm placed above the right forearm and the two forearms now touching each other (see Fig 1). (*ii*) We used only one stimulus configuration with equal horizontal and vertical stimulator distances of 50% total forearm length, resulting in an AR = 1. (*iii*) The stimulation pattern was altered, compared to the previous experiments, in order to keep the direction of motion perception ambiguous. The two stimulators from one forearm were now active in synchrony and in alternation with the two stimulators from the other forearm (see also Fig 1), in order to allow horizontal and vertical motion perceptions (expressed in exogenous coordinates).

### Results

The mean percentage of percepts other than vertical or horizontal motion was 0.5 ± 0.5%.

**Fig 4:**
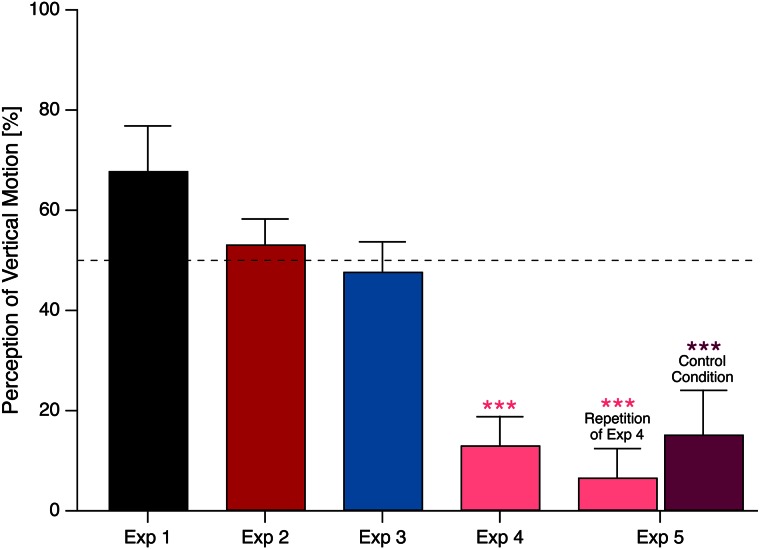
Comparison of %vertical motion perceptions from the five experiments. Percentage of vertical motion percepts, selectively for the conditions with AR = 1 (i.e. equal within- and between-forearm stimulator distances) across all 5 experiments. The horizontal dotted line indicates chance level, i.e. equal probability for vertical and horizontal motion percepts. A weak vertical bias is indicated (but non-significant) in Exp. 1. Overall motion perception stayed ambiguous for Experiments 2 and 3 but was strongly biased towards horizontal motion in Experiments 4 and 5 with 45°-rotation of the forearms. Error bars indicate SEMs. *** indicates p<0.001 (corrected).

With this crossed-forearm configuration we found a highly significant preference for horizontal motion (87% ± 6%, p = 1.9•10^−6^), which is in contrast to the results from all three previous experiments (see Fig 4).

### Discussion

Rotating the forearms by only 45° (instead of 90° as in Experiment 3) completely changed the pattern of tactile motion perception results. We found a strong perceptual bias towards horizontal motion perception (≈ 87%). It was necessary to change the pattern of stimulation in this experiment in order to keep the stimulus ambiguous and thus to allow for both horizontal and vertical motion perception (see Methods and Fig 1), which clearly complicates comparisons with previous experiments. But this hardly explains these unexpectedly clear results and a conclusive explanation is difficult.

**Fig 5:**
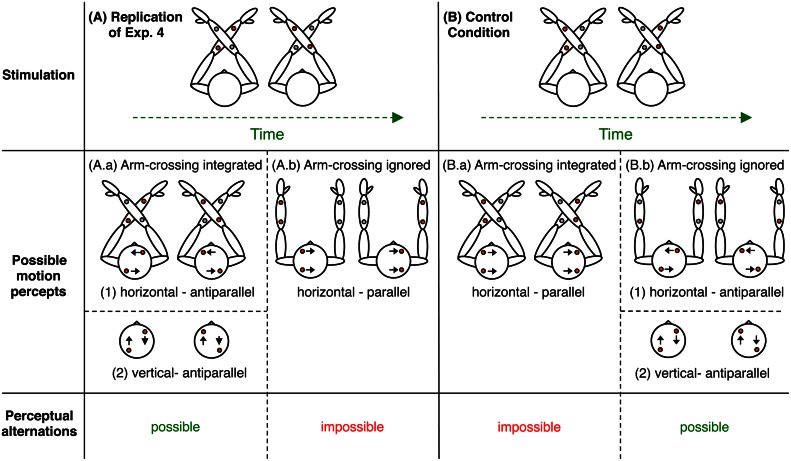
Tactile stimulation modes and possible motion percepts. Top: the representation of the two different tactile SAM stimulation modes from Experiments 4 and 5. Middle: the possible motion percepts, with horizontal or vertical direction and the relation of the perceived motion directions of the two dots’ streams (parallel or antiparallel). Bottom: Indication of whether spontaneous perceptual alternations are possible, given the perceived arm configuration and perceived motion direction.

There are two possible interpretations of these surprising results:

(*i*) The perceptual system takes the crossed forearms into account (Fig 5A.a). If so, the tactile stimulation should evoke the perception of two dots moving back and forth in opposite (i.e. antiparallel) directions and both horizontal and vertical motion perceptions are in principle possible. The reason for the strong horizontal bias in our data, is unclear, however.

(*ii*) The perceptual system ignores the forearm-crossing (Fig 5A.b). Then the tactile stimulation should evoke the perception of two dots moving left and right in parallel. In this case only horizontal motion perception would be possible, which would indeed explain our results.

In order to control for the two possibilities, we asked six of our participants after the experiment about their percept with respect to the two options. Three of them perceived antiparallel horizontal motion, which indicates that the crossing of the forearms was integrated in the perceptual construct (Fig 5A.a). The other three participants reported parallel horizontal motion, which indicates that the arm crossing was ignored during perceptual construction (Fig 5A.b).

## Experiment 5: Crossing Forearms, Variant 2

### Background

A forearm rotation of only 45° provided the strongest perceptual disambiguation effect on tactile SAM motion perception. However, in contrast to the previous results and to our surprise, perception was biased towards horizontal motion. In order to further understand this puzzling result from Experiment 4, we added Experiment 5 with two conditions. In Experiment 4 we had changed the order of tactile stimulation from Experiments 1–3, in order to allow for two possible perceptual interpretations of motion direction and thus keep the stimulus ambiguous. In Condition 1 of Experiment 5, we simply replicated Experiment 4, whereas in Condition 2, we kept the crossed arms but stimulated in the same order as in Experiments 1 to 3. We would like to acknowledge one anonymous reviewer for this suggestion.

### Participants

6 participants (2 males, 4 females; age range 24–32, mean age = 27.5) participated in Experiment 5. Five participants were right-handed and one was left-handed. No participant had participated in Experiments 2; two had participated in Experiment 3 and three had participated in Experiment 4.

### Tactile SAM stimuli and Paradigm

Experiment 5 consisted of two conditions: Condition 1 was a replication of Experiment 4: The two stimulators from one forearm were active in synchrony and in alternation with the two stimulators from the other forearm.

Condition 2 served as control to Condition 1: Here the stimulator closest to the hand of one arm was active in synchrony with the stimulator closest to the crook of the other arm and vice versa. This stimulation mode corresponds to that of Experiments 1–3.

Like in Experiment 4, we tested only one configuration with equal between- and within-forearm stimulator distances at 50% of the total forearm length.

### Results

In Condition 1 we again found a highly significant preference for horizontal motion (94% ± 7%, p = 5.8•10^−6^), which confirms the results from Experiment 4 (see Fig 4 and Table 4). Five out of six participants reported parallel motion perception, indicating that the information about the crossed arms was not integrated into motion processing. Additionally, only one participant indicated antiparallel motion perception.

**Table 4:** Results from Experiment 5.

	Condition 1 (Replication of Exp. 4)		Condition 2	
Participant	Motion Percepts	Reports	Motion Percepts	Reports
A	100% horiz.	parallel	100% horiz.	antiparallel
B	100% horiz.	parallel	95% horiz.	antiparallel
C	100% horiz.	parallel	100% horiz.	parallel
D	95% horiz.	parallel	52% horiz.	both
E	100% horiz.	antiparallel	100% horiz.	both
F	65% horiz.	parallel	61% horiz.	antiparallel

In Condition 2, where we used the stimulation order from Experiments 1–3, we found a similarly strong preference for horizontal motion (85% ± 9%, p = 9.2•10^−4^). Three out of six participants reported having always had antiparallel motion percepts. Two participants reported having had sometimes antiparallel and sometimes parallel motion percepts. Only one participant reported having had always parallel motion percepts. The participants’ perceptual reports from Experiments 5 are summarized in Table 4.

### Discussion

Condition 1 of Experiment 5 replicated the surprising results of close to 90% horizontal motion percepts from Experiment 4. We had to change the stimulation order in Experiment 4 compared to Experiments 1–3, in order to keep the tactile stimulation ambiguous. In the present Experiment 5, we repeated Experiment 4 and added a second condition with crossed arms but the stimulation order from Experiments 1–3. To our surprise, the strong horizontal bias remained. The two conditions from Experiment 5 help to understand the puzzling results from Experiment 4 at least partially, if we postulate the following: the perceptual system of some participants always or sometimes ignored the crossed forearm position during motion perception, even though the crossed forearms touched each other.

The ignorance of the forearm-crossing and the alternative assumption of parallel arms implies horizontal motion as the only interpretation of the tactile stimulation in Condition 1. The two dots will then be perceived as moving in parallel as illustrated in (Fig 5A.b). This is what was reported by participants A, B, C and D in Condition 1 (see Table 4). The ignorance of the forearm-crossing together with the stimulation mode from Condition 2 would lead to motion ambiguity, i.e. both horizontal and vertical motion percepts are possible, but the two dots should be perceived as moving antiparallel as illustrated in (Fig 5B.b1 and 5B.b2). Participants A and B both report almost uniquely horizontal-antiparallel motion perception in Condition 2. Participant B also indicates some alternations of perceived motion direction in Condition 2, as above predicted.

An interesting observation of Experiment 5 is the existence of two types of perceptual alternations. First, participants’ percepts can alternate between horizontal and vertical motion perception. Second, participants motion processing can alternate between a mode *with ignorance of forearm-crossing* and a mode *with integration of forearm-crossing*. Participants C, D may be candidates for the latter observer type: Both seemed to ignore forearm-crossing in Condition 1 and thus reported 95% and 100% parallel-horizontal motion percepts. Participant C further reported also 100% parallel horizontal-motion percepts in Condition 2, which is only possible if he integrated the crossed-forearms in his motion processing, as illustrated in (Fig 5B.a).

Interestingly, Participant D reported 52% horizontal motion percepts with both parallel and antiparallel motion percepts in Condition 2. This is only possible if the participant alternated between integration of the forearm-crossing and ignorance of the forearm-crossing within one experimental condition, as illustrated in (Fig 5B.a and 5B.b). Similarly, the results from Participant E can only be explained if we assume alternations between integration (results from Condition1) and ignorance of forearm-crossing (results from Condition 2).

Participant F's reports from Condition 2 also nicely fit to our explanation. He perceived horizontal and vertical antiparallel motion and thus must have ignored the forearm-crossing as illustrated in (Fig 5B.b1 and 5B.b2).

The only exceptional data in Experiment 5 are from Participant F in Condition 1, where he reported vertical and horizontal parallel motion percepts. Vertical parallel motion perception is not possible from our line of reasoning (see Fig 5A.a.2). Whether Participant F erroneously pressed the wrong button will stay unresolved.

In summary, our postulate of two types of perceptual alternations—concerning motion direction and concerning integration or ignorance of forearm crossing—and different observer types, explained about 92% of the data.

In the present experiment the parallel or antiparallel motion perception was only reported at the end of the measurement, because Experiment 5 was aimed as a control to Experiment 4 and we tried to keep everything beyond the presentation mode identical. These types of tactile stimulation experiments with crossed forearms need to be replicated and extended. In particular, participants should report with each presentation both the motion direction and whether they perceived parallel or antiparallel motion.

## General Discussion

In the present study we analyzed motion perception instability of tactile versions of von Schiller’s [4] SAM motion stimulus as a function of stimulus ARs (i.e. the ratio between horizontal vertical dot distances) with a specific focus on the relation between the two reference frames involved in tactile motion perception.

Tactile SAM stimulation, with vibrators attached to the participants forearms, evoked tactile apparent motion percepts, with similarities to the visual modality: In both modalities motion perception gets unstable at certain stimulus ARs. Further, there is a general preference/bias towards vertical motion perception (i.e. motion from and to the body in the tactile modality). We also found differences between visual and tactile apparent motion perception. It is well known that decreasing ARs strongly biases visual SAM perception to horizontal motion, whereas tactile SAM motion perception stays ambiguous even for very small ARs. Increasing visual SAM ARs strongly biases visual perception towards vertical motion. We also found a vertical bias for the tactile SAM, however, the effect was much weaker (< 70%) than in the visual modality (> 90%), even with very large tactile ARs up to 8.

### About the differences between modalities

Our tactile SAM experiments differ necessarily in a number of aspects (stimulus size, timing, frequency of point alternations, etc.) from those in the visual modality, which may explain the differences in results. However, several modifications of visual SAM experiments did not alter the strong perceptual biases at extreme AR values, e.g. [19,34,35], indicating the robustness of this effect.

An alternative explanation for the differences between visual and tactile apparent motion results may be, that tactile motion perception requires the alignment of the information from the two reference frames, whereas in vision there is only one. At certain stimulus configurations the two reference frames may provide contradictory information, probably destabilizing perceptual constructs. The results from Experiments 1 and 2 point in this direction. We further studied this potential explanation in Experiments 3–5 by rotating the forearms and thus changing the alignment between exogenous and endogenous reference axes. To our surprise, a 90° forearm rotation still supported the primary results: we again found a broad AR range with perceptual instability, a weak bias for horizontal motion, but no bias at all for horizontal motion. Finally, and even more surprisingly, a ±45° forearm rotation evoked a strong horizontal bias (> 87%) even in a configuration with an AR = 1. These findings question the conflict of reference frames as general explanation for the difference between visual and tactile apparent motion perception.

### About the similarities between modalities

There are numerous stimulus examples for perceptual ambiguity both within and between modalities. One important question is whether all these different examples share some common principles and potentially also common neural sources, a possibility discussed at length in previous accounts [11,36]. This refers to the old and still vivid bottom-up vs. top-down debate of whether ambiguity is resolved at lower sensory (bottom-up), or at higher cognitive levels (top-down) beyond sensory modalities. For reviewed, see [37,38]. The present SAM stimuli are well suited to test this generality assumption. The most obvious commonality between visual and tactile motion ambiguity we found, was a general preference for vertical motion (expressed in exogenous coordinates), even though the effect in the tactile modality was much weaker than in the visual modality. Is this preference for vertical motion controlled by a system beyond sensory modalities, which would be in accordance with the top-down approach? And why should such a system prefer vertical and not horizontal motion?

One prominent explanation for the vertical motion bias in the visual modality refers to *inter-hemispheric integration hypothesis* [21,22,39,40]. In short, this hypothesis maintains that, because the right and left visual hemifields are processed in the contralateral brain hemispheres, vertical motion is processed within hemispheres, while horizontal motion needs the interplay of hemispheres. Processing horizontal motion is thus more difficult and less preferred as a solution during perceptual disambiguation.

The same reasoning may apply to tactile motion perception. The endogenous reference frame is most probably based on the somatotopic body representation in the somatosensory cortex. It is known that the left forearm is represented in the right hemisphere and vice versa, e.g. [41]. Perceived within-forearm motion should thus be processed within hemispheres whereas between-forearm motion would require the correspondence between hemispheres. The exogenous reference frame is most probably shared with the visual modality [42] and thus again, what we called perceived vertical motion should have been processed within hemispheres and perceived horizontal motion should have needed the interplay between hemispheres. This reasoning might help to explain the finding of a (weak) tactile vertical bias in Experiments 2 and 3. However, it is difficult to reconcile this explanation with the results from Experiment 3. After ±90° rotation of the forearms, both horizontal and vertical motion directions need both hemispheres and no preference for one motion direction should be observed. However, the patterns of results stay unchanged concerning the weak vertical bias. It is even more difficult to reconcile the results from Experiments 4 and 5 (forearm rotation of ±45°) with the inter-hemispheric integration theory: Motion integration with respect to the endogenous reference frame needs inter-hemispheric integration for both motion directions. For the exogenous reference frame inter-hemispheric integration in the crossed-arm condition is only necessary for horizontal motion. Again, assuming inter-hemispheric communication as more effortful, at least a slight vertical bias would be expected. But we found the opposite, namely a horizontal motion bias, which was much stronger (87%) than all previous tactile vertical motion biases, a finding that we replicated in Experiment 5 with a new set of participants.

Another important factor may be our everyday experience with gravity, which probably more often produces a vertical trajectory on our retinae and thus may, due to long-term memory, *a priori* bias motion perception in the case of ambiguous sensory evidence. Impressive examples of how gravity influences perception can be found in Clément & Demel [43] and Clément & Wood [44].

For the visual SAM, where only one reference system is available, gravity or the inter-hemispheric integration approach or even both may explain the vertical motion bias. For the tactile SAM the perceptual system has to adjust the information from two reference frames and evidences from those can become contradictory. Such a situation may increase the range of stimulus parameters for instability of motion perception, as found for the tactile stimuli. Top-down factors, like experience with gravity, may then tip the balance between interpretations in one or the other direction. This, however, cannot explain the strong horizontal bias, found in Experiments 4 and 5.

In summary, it is neither clear why tactile apparent motion perception stays ambiguous for a broad range of ARs, nor what constitutes the tactile vertical motion bias. It is also unclear whether this weak vertical bias shares some causal mechanisms with the strong visual vertical SAM motion bias and whether inter-hemispheric integration is the mainly relevant factor or only one of several influencing factors. The sudden and strong disambiguation of the otherwise highly ambiguous tactile motion perception in a crossed-forearm condition in Experiments 4 and 5 is clearly the most puzzling finding of the present study and further studies are necessary to understand this phenomenon.

## Conclusions

Overall, our present data are good examples of the general perceptual inference problem [45,46]. The availability of information from the world around us is restricted by the capacities of our senses. The available information is thus inherently incomplete and to a varying degree ambiguous, even though we normally do not notice this. Every available bit of information has potential relevance and enters into a (Bayesian) probability calculation, necessary to construct stable and reliable percepts and thus to solve the perceptual inference problem [45,46]. In the case of tactile apparent SAM motion percepts, our results suggest that there is no fixed rule or relevance hierarchy for the weighting of bottom-up and top-down evidence. Rather, as evident from the differential outcomes of Experiments 1 and 2 on one hand and Experiment 3 and in particular Experiments 4 and 5 on the other hand, the perceptual outcome depends on the current context and in particular on the quality of available information at each perceptual moment. It also depends on the perceptual memory content and probably long-term perceptual statistics (e.g. gravity), as well as on the psychological state and individual perceptual strategy of the observer. The brain architecture with its two hemispheres may also play a role. In the case of sensory ambiguity, slight changes of the sensory input can evoke sudden and fundamental perceptual changes, like a change from a weak vertical to a strong horizontal motion bias, when we change the forearm rotation angle by only ± 45° (Experiments 4 and 5 compared to Experiments 1–3). Our perceptual system is flexible enough to quickly and fundamentally change perceptual interpretations, which may have been one major advantage during evolution.
